# Now more than ever: building a resilient public health future through inclusive leadership

**DOI:** 10.3389/fpubh.2025.1642510

**Published:** 2025-08-11

**Authors:** Laura Magaña, Georges C. Benjamin

**Affiliations:** ^1^Association of Schools and Programs of Public Health, Washington, DC, United States; ^2^American Public Health Association, Washington, DC, United States

**Keywords:** public health leadership, public health education, academic public health, inclusive public health leadership, public health leadership training

## Abstract

This paper explores the urgent need for an inclusive model of leadership in public health, particularly in the context of accelerating social and political change. Drawing on lessons from the COVID-19 pandemic and other recent public health crises, the paper argues that traditional top-down leadership structures—often disconnected from the communities most impacted—are insufficient for responding to modern challenges. Instead, public health systems must invest in cultivating leaders who are embedded in, trusted by, and responsive to diverse populations. Citing workforce declines, public mistrust (1), and the harassment of health officials (2) before, during, and after the COVID-19 pandemic, this paper calls for a national leadership training agenda that spans the academic-to-practice continuum and emphasizes mentorship, flexible educational models, and integration with public health jurisdictions and community-based organizations. The paper also recommends reforming leadership metrics to prioritize measurable impact over positional authority. Ultimately, this paper positions adaptive, equity-focused leadership development as foundational to strengthening the nation’s public health infrastructure. It offers a forward-looking, inclusive vision that aligns leadership development with the evolving realities and demands of 21st-century public health.

The pace of change has never been faster. Whether a public health emergency, political shift, or cultural reckoning, events unfold rapidly and often without warning. The COVID-19 pandemic illustrated this reality as communities across the nation suddenly faced shortages of diagnostic tests and vaccines, limited healthcare capacity, and unreliable information and guidance. In times of crisis, every moment is critical, and effective leadership is paramount in such moments of change.

Effective and trusted leadership is a critical determinant of whether rapidly evolving circumstances yield coordinated action and community resilience. In times of change, people and communities seek direction. They look for leaders who demonstrate competence, compassion, and clear communication—individuals capable of understanding complex systems and responding decisively. Such leaders do not emerge spontaneously. Rather, they require environments that foster their training and development and systems that recognize and support their emergence.

The need for public health leadership training and development is underscored by recent challenges faced by the public health workforce. Pre-COVID-19 workforce declines left gaps in institutional knowledge and experience, and the 2021 Public Health Workforce Interests and Needs Survey exposed an exodus of state and local public health employees, with many departures attributed to inadequate preparation for leadership roles and the escalating demands of public health emergencies (1, 2).

The transition to leadership positions often necessitates new skill sets, a well-documented need among scientific staff and subject matter experts in governmental public health. Compounding this issue is the rise in pandemic-related workplace harassment. Public health officials have faced harassment and threats, contributing to a hostile work environment and further attrition (3). This climate underscores the necessity for leadership training and development that prepare leaders to manage crises, communicate effectively under pressure, and support their teams and communities through adversity.

Within the field of public health, leadership has historically been concentrated among staff supervisors who lack connection to the communities disproportionately affected by health crises. This top-down leadership model is inadequate in addressing the public health needs of a rapidly changing society. To strengthen public health systems, it is essential to cultivate and mentor leaders from a broad range of backgrounds, sectors, and communities. Leadership should not be exclusively defined by formal education or professional status. Public health leadership encompasses mobilizing communities, communicating effectively and empathetically, making informed decisions under pressure, and fostering trust across different populations. These competencies can be developed through intentional investment in training, mentorship, and community engagement.

To ensure a more resilient public health infrastructure, there must be a national commitment to preparing individuals across all communities to become public health leaders. This includes not only public health practitioners, but also individuals who hold trust and influence within their communities. Strengthening public health requires a locally grounded leadership model that is adequately supported through academic and practice partnerships.

A national public health leadership training agenda, one that is comprehensive enough to address systemic challenges while remaining adaptable to the diverse contexts in which public health operates, should support leadership development across the academic and practice continuum, from degree to service. It should support integrating leadership training into classrooms, fund mentorship and fellowship programs for emerging leaders, and establish infrastructure to facilitate interdisciplinary and cross-sector collaboration.

Public health leadership training and development should be responsive to the needs of all communities, as well as to the distinct operational capacities of state, Tribal, local, and territorial health departments, health systems and health clinics, community-based organizations, and academic institutions. Flexibility and contextual sensitivity are essential to building a more resilient and equitable public health system. Moreover, we must establish metrics by which we assess leadership effectiveness, which should not be measured solely by visibility or positional authority, but by tangible impact. Key indicators might include improved public understanding of health issues, adoption of equitable policies, and increased community engagement and empowerment. Achieving these outcomes will require leaders who are not only well-prepared but also deeply connected to the populations they serve.

Implementing a national public health leadership training agenda will not be without challenges. Coordinating efforts across academic institutions, health departments, and community organizations requires alignment around shared goals, metrics, and definitions of effective public health leadership, which can be a complex and time-consuming effort requiring sustained commitment from multiple stakeholders. Further, variability in resources, infrastructure, and priorities across jurisdictions can hinder the consistent implementation of training programs. To overcome these obstacles, it will be critical to allow for local flexibility, ensure sustainable funding, and promote cross-sector collaboration and accountability.

As the pace of change accelerates, so too must our approach to public health leadership. Meeting the challenges of the future requires leaders who are prepared to navigate uncertainty, respond swiftly in times of crisis, and maintain public trust. This calls for investment in leadership training and development that is aligned with the realities of an evolving public health landscape. By focusing on sustained training, mentorship, and systems-level support, we can strengthen the capacity of our public health infrastructure to respond effectively to whatever challenges lie ahead. The path forward is clear: we must equip our public health leaders with the tools they need to act decisively and uphold the health and well-being of our nation.

## Data Availability

The original contributions presented in the study are included in the article/supplementary material, further inquiries can be directed to the corresponding author.
